# Heat map visualization for electrocardiogram data analysis

**DOI:** 10.1186/s12872-020-01560-8

**Published:** 2020-06-08

**Authors:** Haisen Guo, Weidai Zhang, Chumin Ni, Zhixiong Cai, Songming Chen, Xiansheng Huang

**Affiliations:** 1 Department of Cardiology, Shantou Central Hospital, Shantou, 515000 Guangdong China; 2 Department of Cardiology, the First Affiliated Hospital of Shantou University Medical College, No. 57 Changping Road, Shantou, 515000 Guangdong China

**Keywords:** Heat map, Visualization, Electrocardiogram, Data analysis

## Abstract

**Background:**

Most electrocardiogram (ECG) studies still take advantage of traditional statistical functions, and the results are mostly presented in tables, histograms, and curves. Few papers display ECG data by visual means. The aim of this study was to analyze and show data for electrocardiographic left ventricular hypertrophy (LVH) with ST-segment elevation (STE) by a heat map in order to explore the feasibility and clinical value of heat mapping for ECG data visualization.

**Methods:**

We sequentially collected the electrocardiograms of inpatients in the First Affiliated Hospital of Shantou University Medical College from July 2015 to December 2015 in order to screen cases of LVH with STE. HemI 1.0 software was used to draw heat maps to display the STE of each lead of each collected ECG. Cluster analysis was carried out based on the heat map and the results were drawn as tree maps (pedigree maps) in the heat map.

**Results:**

In total, 60 cases of electrocardiographic LVH with STE were screened and analyzed. STE leads were mainly in the V_1_, V_2_ and V_3_ leads. The ST-segment shifts of each lead of each collected ECG could be conveniently visualized in the heat map. According to cluster analysis in the heat map, STE leads were clustered into two categories, comprising of the right precordial leads (V_1_, V_2_, V_3_) and others (V_4_, V_5_, V_6_, I, II, III, aVF, aVL, aVR). Moreover, the STE amplitude in 40% (24 out of 60) of cases reached the threshold specified in the STEMI guideline. These cases also could be fully displayed and visualized in the heat map. Cluster analysis in the heat map showed that the III, aVF and aVR leads could be clustered together, the V_1_, V_2_, V_3_ and V_4_ leads could be clustered together, and the V_5_, V_6_, I and aVL leads could be clustered together.

**Conclusion:**

Heat maps and cluster analysis can be used to fully display every lead of each electrocardiogram and provide relatively comprehensive information.

## Background

Electrocardiography, a non-invasive and economical detection method, is widely used in clinical practice [1, 2]. An electrocardiogram (ECG) contains multiple lead data, and the combinations of different ECG lead shifts have different clinical significance [2, 3]. Therefore, in clinical research, it would be helpful for readers to analyze, understand and memorize the data and research conclusion by showing the individual ECG results for each lead [4]. Nevertheless, for the moment, most clinical ECG studies still take advantage of traditional statistical functions, and their results are mostly presented by traditional charts, such as tables, histograms, and curves. Traditional charts have difficulty fully showing the details of each case, and only briefly quantify the proportion of target changes and the average value of the changes. Moreover, traditional charts cannot show data intuitively, which is not conducive to a synthesized understanding of the results [5].

A heat map is a graphical representation of data that simultaneously reveals the row and column hierarchical cluster structure in a data matrix [6]. Different colors are used to represent the magnitude of a certain monitoring value. By virtue of the inherent sensitivity of human beings to color, heat maps transform data into a color summary, which makes the distribution and characteristics of the data clear at a glance and makes it easy to distinguish and summarize abnormalities [6, 7]. Moreover, individual data points can be grouped together based on their corresponding heat map color [6, 7]. In all, a heat map conveys complex data concisely and completely at a single glance.

At present, heat maps are mainly used to display genome sequencing results [8]. We found that the structure of ECG data is similar to the structure of genome sequencing data. Each subject has a large number of observed values (leads vs. genes), and the observed values are quantitative (amplitude vs. gene expression level). Their high level of similarity in data structure suggests that heat maps can be applied to visualization of ECG data. Therefore, we analyzed and displayed data for left ventricular hypertrophy (LVH) with ST-segment elevation (STE) by a heat map in order to explore the feasibility and clinical value of heat map visualization and representation of ECG data. Our hypothesis was that heat maps could be used to fully display each lead of each ECG, and cluster analysis of in heat maps could help discover the potential association between different ECG leads.

## Methods

We sequentially collected the electrocardiograms of inpatients in the First Affiliated Hospital of Shantou University Medical College from July 2015 to December 2015 in order to screen the cases of LVH with STE. Inclusion criteria included: 1) an ECG that conformed to Sokolow-Lyon criteria: R_V5_ + S_V1_ ≥ 4.0 mV (males), R_V5_ + S_V1_ ≥ 3.5 mV (females) [9]; 2) an ST-segment elevation that appeared in any ECG lead and an elevation amplitude greater than 0.05 mV [10]; 3) patient age older than 18 years. Exclusion criteria included confounding factors that may affect the ST-segment, such as myocardial infarction, ectopic rhythm, pacemaker implantation, myocarditis, pericarditis, congenital heart disease, or cardiomyopathy [11, 12]. Relevant ECG features, including heart rate, rhythm, STE and T waveform, were collected by cardiologists. For ECGs with STE, the magnitude of elevation was further evaluated [13]. Continuous variables are reported as mean (SD) or median (IQR), as appropriate. Categorical variables are described with absolute and relative frequencies. The study was approved by the ethics committee of Shantou University Medical College.

### Drawing the heat map

First, HemI 1.0 software (Huazhong University of Science and Technology, Hubei, China) was used to draw a heat map for displaying STE in each lead of each collected electrocardiogram [8]. HemI software used a red, green, and blue tricolor in a 256-color mode. The inputted ST-segment data were linearly normalized as below:

$$ NV=\frac{OV-\mathit{\operatorname{Min}}}{\mathit{\operatorname{Max}}-\mathit{\operatorname{Min}}}\times 256\times 3 $$

where

*NV* = normalized value;

*OV* = original value;

*Max* = the maximum of all OVs;

*Min* = the minimum of all OVs.

The transverse axis of the heat map represented the leads of the ECG and the longitudinal axis represented the cases. Each lattice color represented an ST-segment condition, with blue representing a normal or depressed ST-segment, red representing an elevated ST-segment, and the lattice color changing from blue to red representing the extent of increase in ST-segment amplitude. Second, we analyzed the ECG leads whose elevation amplitude reached the STE threshold specified in the ST-segment elevation myocardial infarction (STEMI) guideline and then displayed them in a heat map. Each lattice color represented an STE condition. Red expressed an ST-segment magnitude meeting the thresholds specified in STEMI guidelines, while blue expressed an ST-segment magnitude that did not meet the STEMI criteria for STE.

### Conducting cluster analysis

We also conducted a cluster analysis based on the heat map to discover the association of ECG lead shifts. The results of cluster analysis were drawn as tree maps (pedigree maps) in the heat map. Conventionally, the tree map was attached to the heat map and could be considered part of a heat map.

Cluster analysis techniques are classified as hierarchical and nonhierarchical clustering algorithms. The choice of a cluster analysis techniques depends on the nature of variables. In the initial step, two objects with the lowest distance are combined into a cluster. In the next step, the remaining objects are analyzed one by one, and each object is classified into the previous existing cluster or a new cluster according to its distance from the analyzed objects. The process is repeated until each object has been identified and allocated to a specific cluster. Three types of linkage criteria (average linkage clustering, minimum linkage clustering, and maximum linkage clustering) can be adopted for the hierarchical clustering algorithms (Supplementary Table S1) [8]. The most appropriate clustering algorithm that turn out to be the most meaningful for a particular application is chosen. In this study, average linkage clustering was used to conduct a cluster analysis (Supplementary Table S1) [8, 14].

### STEMI guidelines

Acute coronary artery occlusion was considered in the following cases: STE > 0.1 mV in all leads other than leads V_2_-V_3_, where the following cutoff points apply: STE > 0.2 mV in men ≥40 years; STE ≥0.25 mV in men < 40 years, or STE ≥0.15 mV in women [10]. The ST-segment shift should be present in two or more contiguous leads [15].

## Results

**Fig. 1:**
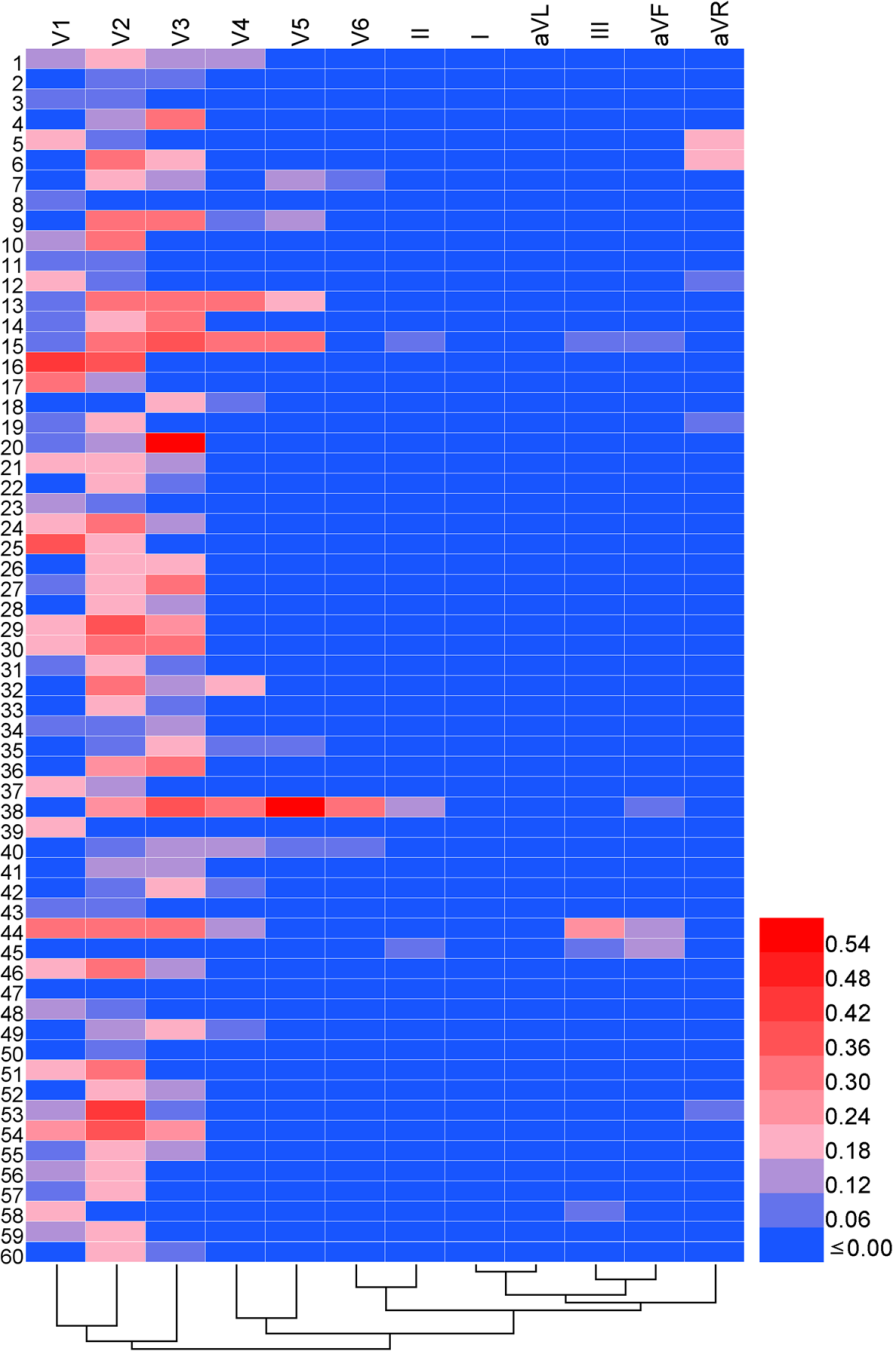
Heat map corresponding to ST-segment elevation conditions for each case. The transverse axis of the heat map represents the leads of the electrocardiograms, and the longitudinal axis represents the cases. Each lattice color represents an ST condition, with blue representing a normal or depressed ST-segment, red representing an elevated ST-segment, and blue-to-red lattice color changes representing the increase in ST amplitude. STE = ST-segment elevation

In total, 60 cases of electrocardiographic LVH with STE were screened and analyzed. We found that the STE changes in these cases appeared mainly in the V_1_, V_2_ and V_3_ leads. The frequency of STE in these leads was 65, 91.7 and 63.3%, and the elevation ranges were 0.15 (0.1–0.2) mV, 0.2 (0.1–0.3) mV and 0.175 (0.15–0.3) mV, respectively (Table 1). Commonly used heat map generation methods were found to be able to generate a heat map and conduct cluster analysis for these results. The STE of each lead of each collected electrocardiogram was displayed in a heat map (Fig. 1). According to the tree map in the heat map, ECG leads were clustered into two categories based on the STE condition, including the right precordial leads (V_1_, V_2_, V_3_) and others (V_4_, V_5_, V_6_, I, II, III, aVF, aVL, aVR) (Fig. 1).

**Table 1:** Characteristics of electrocardiographic LVH with STE

Leads	Number of STEs(n, %)	Amplitude of STEs (mV)	STE characteristic			
			Platform elevation	Bow-back elevation	Upwardly inclined elevation	Concave face up elevation
**Right precordial**						
V_1_	39 (65)	0.15 (0.1–0.2)	31 (51.7%)	2 (3.3%)	6 (10%)	0
V_2_	55 (91.7)	0.2 (0.1–0.3)	29 (48.3)	5 (8.3)	20 (33.3)	1 (1.7)
V_3_	38 (63.3)	0.175 (0.15–0.3)	20 (33.3)	5 (8.3)	11 (18.3)	2 (3.3)
**Left precordial**						
V_4_	12 (20)	0.15 (0.1–0.275)	4 (6.7)	1 (1.7)	4 (6.7)	3 (5)
V_5_	7 (11.7)	0.15 (0.1–0.3)	2 (3.3)	1 (1.7)	1 (1.7)	3 (5)
V_6_	3 (5)	0.1	0	0	0	3 (5)
**High lateral**						
I	0	0	0	0	0	0
aVL	0	0	0	0	0	0
**Inferior leads**						
II	3 (5)	0.1	3 (5)	0	0	0
III	4 (6.7)	0.1 (0.1–0.2125)	4 (6.7)	0	0	0
aVF	3 (5)	0.125 (0.1–0.15)	3 (5)	0	0	0
**Others**						
aVR	5 (8.3)	0.1 (0.1–0.2)	5 (8.3)	0	0	0

Values are median (interquartile range) or n (%)

*LVH* left ventricular hypertrophy, *STE* ST-segment elevation

**Fig. 2:**
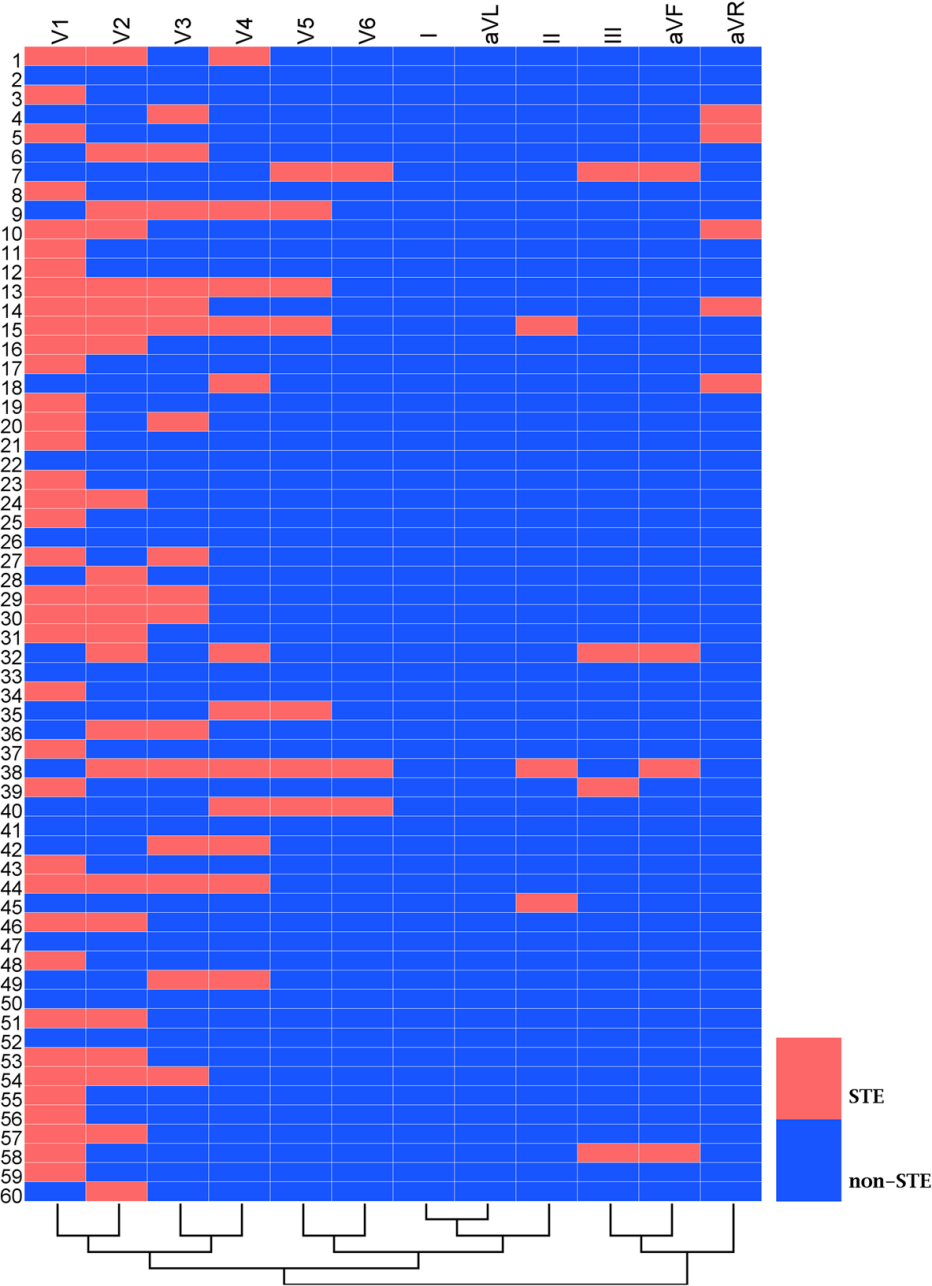
Heat map corresponding to an ST-segment elevation amplitude that reached the threshold specified in the STEMI guidelines. The transverse axis of the heat map represents the leads of the electrocardiograms, and the longitudinal axis represents the cases. Each lattice color represents an STE condition. Red indicates an STE magnitude that meets the thresholds specified in the STEMI guidelines, whereas blue color indicates an ST that did not meet STEMI guidelines for STE. STEMI=ST-segment elevation myocardial infarction; STE = ST-segment elevation

The STE amplitude in some of these cases even reached the threshold specified in the STEMI guidelines [15]. When the STEMI threshold was used to judge the STE, the STE frequencies of the V_1_, V_2_ and V_3_ leads were 61.67, 38.33 and 26.67% respectively. Similarly, we found that commonly used methods could generate a heat map and conduct cluster analysis successfully for these results. According to the heat map cluster analysis, the leads with STE reaching the prescribed threshold could also be clustered into three categories, which showed that the III, aVF and aVR leads could be clustered together, the V_1_, V_2_, V_3_ and V_4_ leads could be clustered together, and V_5_, V_6_, I and aVL leads could be clustered together (Fig. 2).

## Discussion

The traditional text-based data processing and display model has restricted the interpretation of big data in the life sciences. It is extremely difficult for traditional charts to fully display each lead of each ECG within the space of a limited paper. Current electrocardiogram studies just use traditional charts to show the proportion and average changes of each lead, but visualization technology can be helpful for audiences to understand, analyze and memorize information, and also discover important hidden information [16, 17]. A heat map is a commonly used visualization tool, which can provide clues for data mining and has wide prospects for application in the field of medical science [18]. A heat map displays data through color depth, which is intuitive and readable, and can help non-statisticians understand and analyze data [5, 16]. In our study, we tried to apply heat map data visualization technology to display and analyze ECG data.

Previous studies have shown that the incidence of mistaken diagnosis of acute myocardial infarction in “chest pain centers” is relatively high, and the occurrence of mistaken diagnosis events is closely related to electrocardiographic LVH with STE [19–21]. In our paper, we analyze and display data for LVH with STE by a heat map to explore the feasibility and clinical value of heat mapping for ECG data visualization. Using heat maps to display ST- segment elevation data shows the following advantages: (1) Commonly used heat map generation methods could be easily used to generate a heat map for ST- segment elevation data. (2) Compared with traditional tables, heat maps can fully display each lead of each ECG and provide physicians with more comprehensive information. By means of heat map visualization, physicians are able to understand the ECG characteristics of LVH with STE more thoroughly [19, 22]. (3) Showing each lead of each case can help researchers locate the lesion in a specific area of the heart for each individual easily. (4) Moreover, fully displaying each lead aids in discovering combined lesions in different heart areas. (5) The details of atypical combination of lead shifts in rare cases can be shown to audiences in a heat map, which may help reduce misdiagnosis.

In our study, we also used cluster analysis to explore the combination of STE leads in patients with electrocardiographic LVH. We found that the cluster analysis applied to clinical ECG research could help researchers explore the correlation between different leads. It might help researchers locate ECG changes (primary territory affected) more easily and discover atypical combination of leads. In rare cases with atypical lead combinations, traditional methods rely on the subjective judgment of the researchers to identify clinical significance. Researchers often need to classify cases with similar clinical manifestations and then analyze their electrocardiograms to identify atypical ECG lead combination characteristics. However, heat map-based cluster analysis can provide us with an objective classification result and identify hidden interconnections of each lead. Investigators can directly obtain an atypical lead combination and then analyze relevant cases to determine if it is clinically meaningful. This provides a new analytical method for researchers. For example, the cluster analysis in our paper found that III, aVF and aVR leads could be clustered together base on ST-segment conditions in patients with LVH. This finding indicates that one should be very cautious in diagnosing acute inferior myocardial infarction in patients with LVH when STE appears in the III, aVF and aVR leads simultaneously. In summary, cluster analysis in heat map is a powerful tool for discovering the combinations of lead shifts.

Each ECG includes 12–18 leads, and provides quantitative data, such as the amplitude of the ST-segment shift, Q wave depth, and R/S ratio. Traditional charts have difficulty in fully showing such a large amount of data. For instance, a study by Shemirani et al. explored the predictive value of ECG on criminal vessels in patients with acute posterior myocardial infarction [23]. One hundred thirty-eight cases were included in their study. If they wanted to display the data of each lead, data for 2484 leads would need to be displayed, an impossibility for a traditional chart. Thus, their paper just used tables to show the proportion and average magnitude of ECG shifts in each lead. Through reading their paper, readers can only conclude that the frequency of the ST-segment shift in the V_5_, V_6_, I and aVL leads is higher in patients with acute circumflex occlusion, while the frequency of the ST- segment shift in V_1_, V_3R_ and V_4R_ leads is higher in patients with right coronary artery occlusion. However, their paper cannot show the regularity of combination of each lead. In another study, Wu et al. found that J waves in patients with acute myocardial infarction are an independent risk factor for poor prognosis [24]. Nonetheless, this paper could not demonstrate the combination of J wave occurrences in different leads in each case, let alone the amplitude of J waves in each lead, which is not helpful for audiences to identify high-risk cases in clinical practice. These examples illustrate the challenge of traditional charts in displaying ECG results. Previous publication has demonstrated that professional interpreting graphics with missing data tended to misinterpret the results [16]. Viewing more complete graphics may enhance the quality of decision. Hence, a data visualization tool, such as a heat map, may be useful and necessary for these two studies to compare and distinguish each lead shift of each collected cases. The heat map facilitates easy-to-implement visualization of ECG changes and number of changed leads.

Moreover, a heat map can also help to explore the relationship between each lead through cluster analysis. Cluster analysis is a statistical analysis technique, which classifies the research objects into relatively homogeneous groups [25, 26]. Cluster analysis provides additional information to help researchers discover the interconnection of each lead. The results of cluster analysis can be drawn as tree maps in the heat map. The tree graph attached to the heat map shows the entire process of clustering and displays which leads are grouped together from the visual level [8]. Conventionally, the tree map is attached to the heat map and could be considered part of a heat map.

For the moment, most published clinical ECG studies cannot fully display individual ECG data [16, 27]. Heat mapping might be an effective way to solve the difficulty of displaying ECG data to help readers interpret, communicate, and remember the ECG shift trends [5, 16]. Heat mapping may be used to display various features of an electrocardiogram, such as P wave duration, P wave voltage, QRS duration, QRS voltage, P-R duration and QT duration. More importantly, there is no technical threshold to produce heat maps. Our study found that commonly used heat map generation software can generate heat maps and conduct cluster analysis for ECG data without a requirement for special techniques. HemI software, R software, GraphPad prism software, online tools and even Microsoft Excel software can be used to draw heat maps easily and quickly. Therefore, together with utility (functional effectiveness) and usability (perceived ease of use), heat maps can be a useful tool for ECG data analysis. Given the importance of ECG in clinical practice, such visualization may help physicians increase knowledge of ECG and improve the quality when making decisions.

### Limitations

Firstly, compared with traditional tables, heat maps may not be precise enough for reporting quantitative data. However, heat maps can be used as an additional tool to display data more comprehensively and help readers better understand the results. Secondly, in our paper, we applied heat mapping to show STE in LVH cases to evaluate the value of heat mapping in ECG visualization. Since the ECG data came from our ongoing projects, our research objective, data collection and relevant conclusions are incomplete. Nevertheless, the data of LVH with STE are used as demonstration examples to confirm the advantages and feasibility of heat mapping in displaying ECG data. The main purpose of this paper was to use part of the data in our ongoing project to draw heat maps in order to verify the feasibility of using heat maps. More details of LVH findings will be published after our project is completed. Thirdly, the content of this paper may be relatively simple because it only introduces how to apply heat mapping to the visualization of ECG data. However, based on our study, heat mapping to express and analyze ECG data will be evaluated by more and more researchers, which may promote application of heat mapping to clinical ECG research. Moreover, there are several open access databases with large amount of ECG data (STAFF III ECG database, PhysioNet databases, etc.) [28, 29]. Our study may facilitate database administrators and researchers to apply heat maps to display and analyze electrogram data. To the best of our knowledge, this is the first attempt to apply heat mapping to ECG data display.

## Conclusion

Commonly used heat map generation methods could be used to generate a heat map for ST- segment elevation data. Heat maps and cluster analysis can be used to fully display each lead of each electrocardiogram and provide relatively comprehensive information. Heat mapping is a powerful tool for data visualization in clinical electrocardiogram research.

## Supplementary information

**Additional file 1: Table S1.** Optional linkage criteria for the hierarchical clustering.

## Data Availability

Raw data supporting the obtained results are available at the corresponding author.

## Abbreviations

ECG: Electrocardiogram
LVH: Left ventricular hypertrophy
STE: ST-segment elevation
STEMI: ST-segment elevation myocardial infarction
