# Supplementary material for: Heat map visualization for electrocardiogram data analysis
Source: BMC Cardiovasc Disord. 2020 Jun 8;20:277. doi: 10.1186/s12872-020-01560-8 (PMC7281952; doi:10.1186/s12872-020-01560-8)
Supplement: Supplementary file 1 — Additional file 1: Table S1. Optional linkage criteria for the hierarchical clustering. [file 12872_2020_1560_MOESM1_ESM.docx]

**Supplementary Table S1.** Optional linkage criteria for the hierarchical clustering.

| **Linkage criterion** | **Equation** |
| --- | --- |
| Average linkage clustering  (adopted in our study) | $\frac{1}{\left\vert A \right\vert-\vert B\vert}\sum_{a\in A} \sum_{b\in B} d(a,b)$ |
| Minimum linkage clustering | min{d(a, b) : a∈A, b∈B} |
| Maximum linkage clustering | max{d(a, b) : a∈A, b∈B} |

Average linkage clustering, minimum linkage clustering, and maximum linkage clustering are three commonly used linkage criteria to calculate the pairwise distances for the hierarchical clustering. (PloS one 2014, 9(11): e111988.)
